# Supplementary material for: Serial processing of kinematic signals by cerebellar circuitry during voluntary whisking
Source: Nat Commun. 2017 Aug 10;8:232. doi: 10.1038/s41467-017-00312-1 (PMC5550418; doi:10.1038/s41467-017-00312-1)
Supplement: Supplementary file 1 — Supplementary Information [file 41467_2017_312_MOESM1_ESM.pdf]

File Name: Supplementary Information
Description: Supplementary Figures and Supplementary Table

File Name: Peer Review File

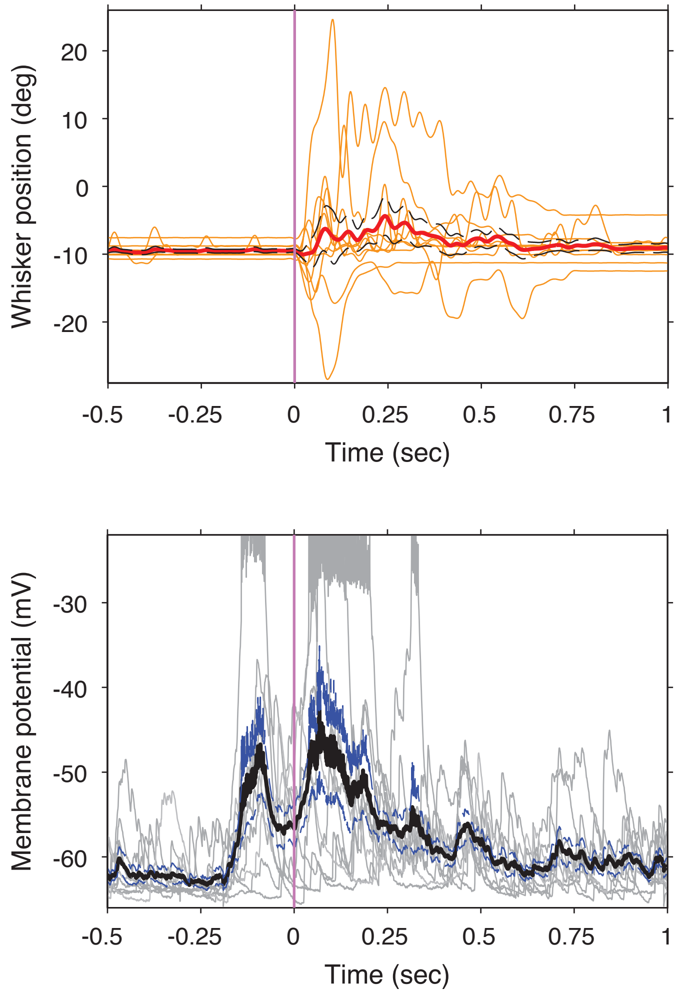

Supplementary Figure 1 - Whisking-related fluctuations in granule cell membrane potential

Top, whisker motion during individual movement epochs (orange traces) aligned to whisking onset (purple line). Red line, black outline: mean whisker position ± S.E.M for all trials.
Bottom, membrane potential (grey traces) for corresponding movement epochs shown above. Black line, blue outline: mean membrane potential ± S.E.M for all trials. GC depolarisation precedes whisking onset.

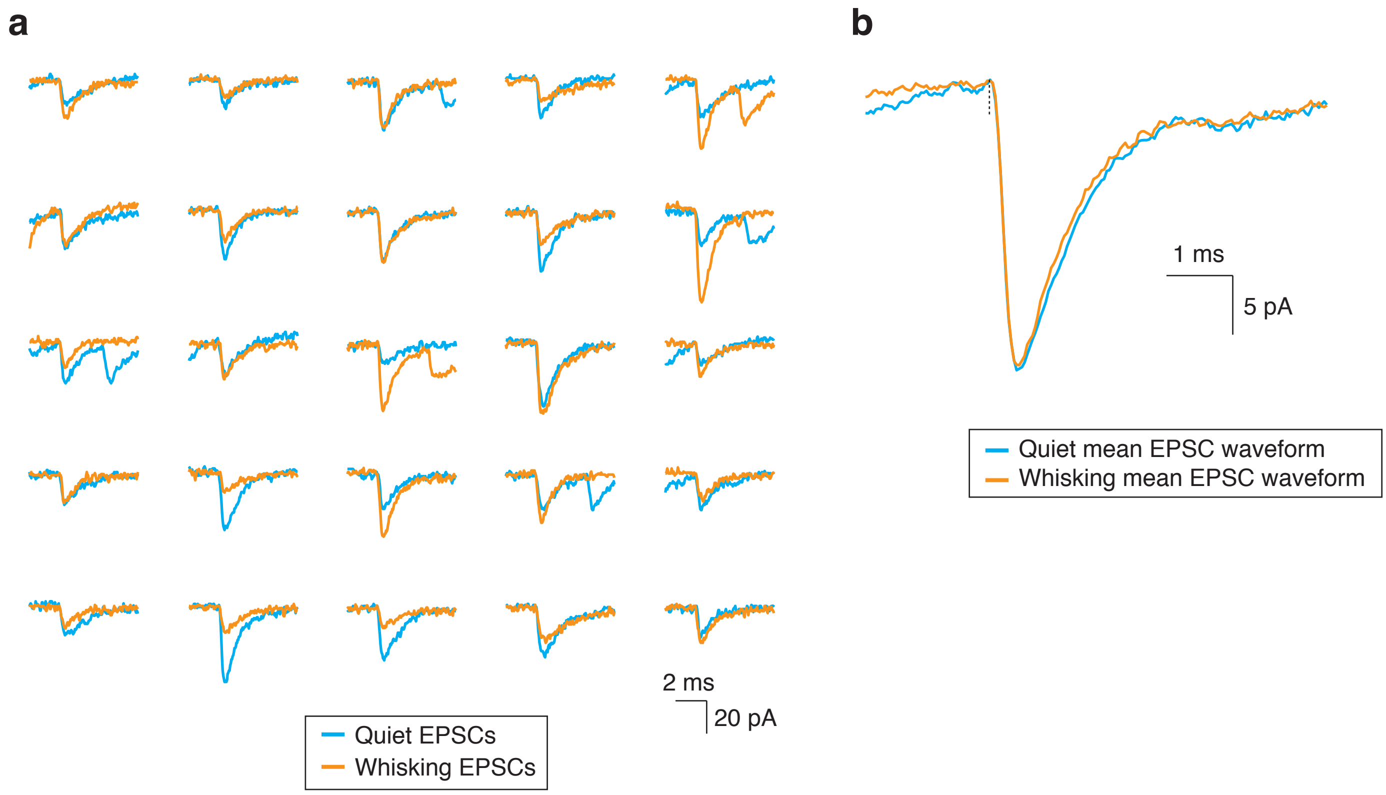

Supplementary Figure 2 - Excitatory postsynaptic currents (EPSCs) recorded from granule cells in awake behaving mice

a, Representative raw EPSC waveforms recorded in voltage clamp mode at -70 mV during epochs of quiescence (blue) and whisking (orange). **b**, Mean waveforms for quiescent (blue) and whisking (orange) epochs reveal no change in the amplitude or time-course of granule cell EPSCs.

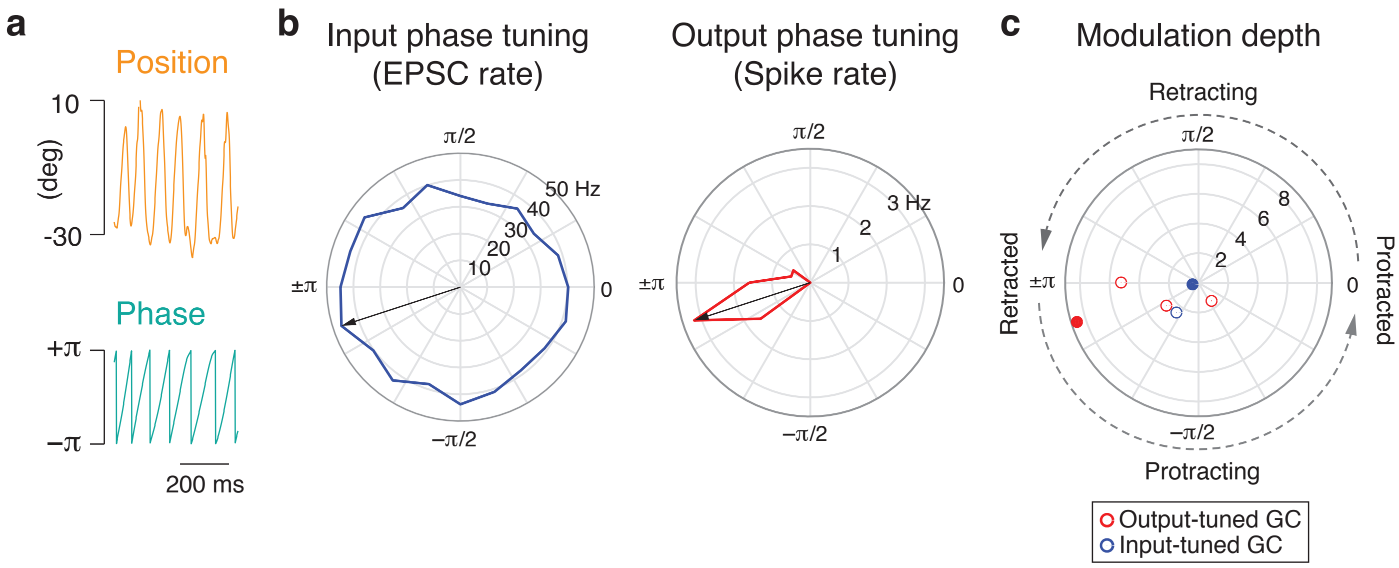

Supplementary Figure 3 - GC tuning to whisking phase

a, Hilbert transform was performed on whisker position traces (orange) to obtain phase of whisking cycle (teal). **b**, Phase tuning of synaptic input (left, blue) and spike output (right, red) for a single GC that exhibited statistically significant tuning ($p < 0.05$, Kuiper's test) in both voltage clamp and voltage recording configuration. Preferred phase (black arrow) was similar for input and output, although output was considerably more selective. **c**, Modulation depth (radius) and preferred phase (polar angle) for all GCs that exhibited significant phase tuning for EPSC rate (blue; n = 2/11) or spike rate (red; n = 4/25). Filled circles indicate example shown in **b**. Input and output tuning were not compared directly in other GCs.

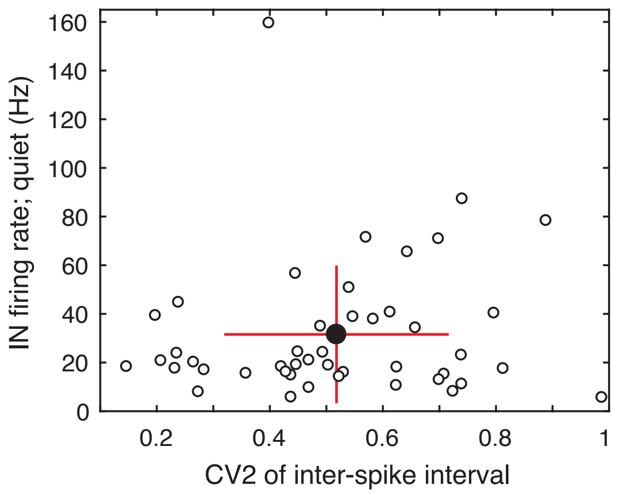

Supplementary Figure 4 - Firing properties of cerebellar interneurons in awake mice

Firing rate and CV2 of all recorded interneurons during quiescence. Filled circle: mean ± standard deviation.

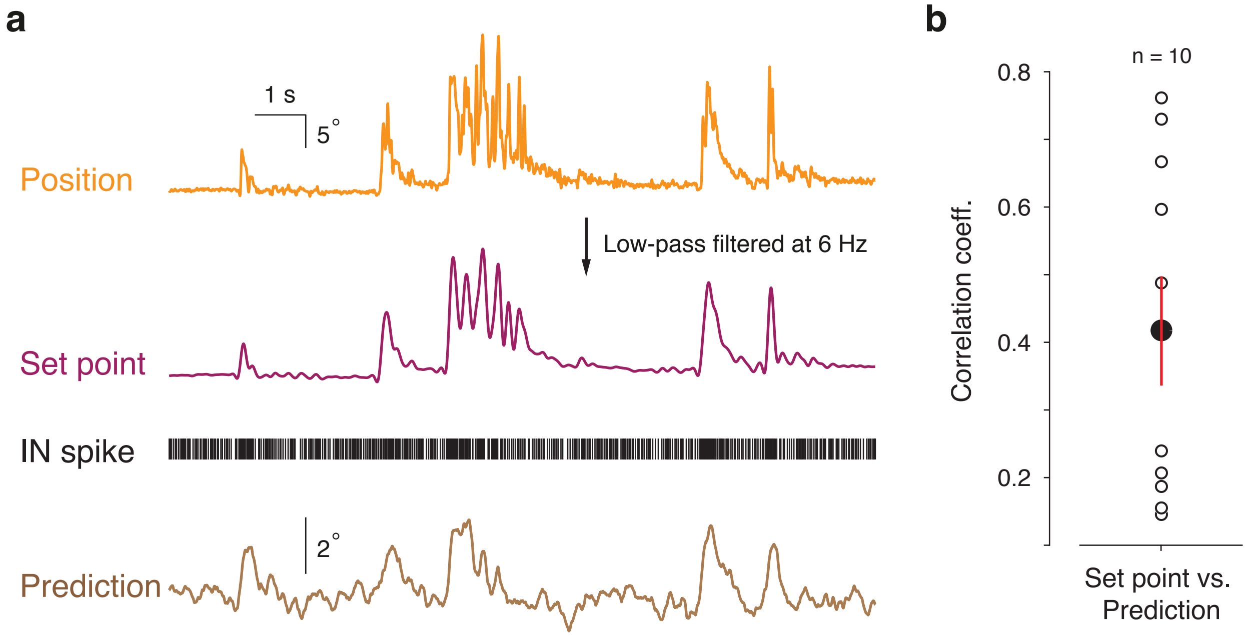

Supplementary Figure 5 - Partial reconstruction of whisker set point trajectory from single interneuron spiking activity

a, Top: whisker position (orange) and the corresponding kinematic parameter set point (purple), derived from low-pass filtering at 6Hz. Bottom: spike train of a single IN and the decoded whisker motion using linear transfer function method (brown trace, see Materials and Methods). **b**, Correlation coefficient values between movement prediction and real set point for all interneurons ($p < 0.05$). Overall, spiking from single INs is sufficient to partially recover real-time fluctuations in set point during movement.

IN No.	R^2	Slope (Hz/deg)	Gain (Hz/deg)	Degrees of freedom	F-statistics	p-value	Direction
1	0.93	7.3	7.3	9	100.4	8.37E-06	bi
2	0.96	-10.6	10.6	3	49.7	0.019541	uni
3	0.99	-18.8	18.8	4	522.2	1.84E-04	uni
4	0.97	20.1	20.1	4	89.9	2.49E-03	bi
5	0.94	-46.5	46.5	5	65.4	1.27E-03	bi
6	1	52.9	52.9	5	2251.9	1.18E-06	bi
7	0.88	-82.8	82.8	10	68.8	1.66E-05	bi
8	0.94	-12.9	12.9	4	44.8	6.81E-03	bi
9	0.96	33.8	33.8	3	47.2	0.020535	bi
10	0.96	6.7	6.7	6	119.5	1.11E-04	uni
11	0.98	31.5	31.5	4	19.5	0.021536	uni
12	0.98	15.4	15.4	7	342.5	1.61E-06	uni
13	0.99	11.4	11.4	7	526.1	4.50E-07	uni
14	0.89	-28.8	28.8	7	46.5	4.88E-04	bi
15	0.97	32.2	32.2	4	120.8	1.61E-03	uni
16	0.97	2.7	2.7	3	62.3	0.01568	uni
17	0.89	41.5	41.5	12	86	1.56E-06	bi
18	1	7.3	7.3	4	146.7	1.21E-03	bi
19	0.97	-10.9	10.9	3	70.8	0.013823	bi

Supplementary Table 1 – Summary of linear regression models for individual interneurons

Linear regression models were fitted on mean whisker position values over a spike rate modulation range of at least 30 Hz (degrees of freedom >= 3). Analysis of variance (ANOVA) was performed for individual linear models, and p < 0.05 for the F statistic was considered statistically significant. IN13 (blue) and IN19 (purple) correspond to examples shown in Figure 7 (a, b), respectively.
